# Seasonal Variations of Low-Molecular-Weight Organic Acids in Three Evergreen Broadleaf Rhododendron Forests

**DOI:** 10.3390/metabo13010119

**Published:** 2023-01-12

**Authors:** Xiaofei Lv, Jiangtao Hao, Yumeng Zhao, Chaochan Li, Wenxuan Quan

**Affiliations:** Guizhou Provincial Key Laboratory for Information Systems of Mountainous Areas and Protection of Ecological Environment, Guizhou Normal University, Guiyang 550001, China

**Keywords:** low-molecular-weight organic acids, subtropical forest, litters, humus

## Abstract

Low-molecular-weight organic acids (LMWOAs) are widely distributed in forests. Fresh leaves, litter, humus, and the topsoil layer of representative *Rhododendron delavayi* (RD), *Rhododendron agastum* (RA), and *Rhododendron irroratum* (RI) in the Baili Rhododendron National Forest Park were sampled to explore their seasonal changes. The contents of oxalic, tartaric, malic, citric, acetic, lactic, succinic, and formic acids in samples from different seasons were determined by high-performance liquid chromatography. The results showed that the composition and content of the LMWOAs in the fresh leaves, litter, humus, and topsoil layer of the rhododendrons were affected by the tree species, samples, and season. The main LMWOA was oxalic acid (the average content in the samples was 195.31 µg/g), followed by malic acid (the average content in the samples was 68.55 µg/g) and tartaric acid (the average content in the samples was 59.82 µg/g). Succinic acid had the lowest content; the average content in the samples was 18.40 µg/g. The LMWOAs of the RD were the highest (the average content in the samples was 517.42 µg/g), and the LMWOAs of the RI were the lowest (the average content in the samples was 445.18 µg/g). The LMWOAs in the three rhododendron forests were in the order of fresh leaves > litter > humus > soil layers. This study showed the seasonal distribution characteristics of LMWOAs in three evergreen broadleaf rhododendron forests, and the results provide a reference for ecosystem management and the protection of wild rhododendron forests.

## 1. Introduction

Low-molecular-weight organic acids (LMWOAs) are ubiquitous on earth. They are important products in the metabolic pathways of organic matter and part of the carbon cycle [1,2]. Acetic, aconitic, citric, fumaric, malic, malonic, and oxalic acid, as well as many other LMWOAs, have been detected in various environmental samples, such as plant organs and rainwater, ice, marine sediments, and soil [3,4,5,6]. LMWOAs are commonly found in forest soils. The main sources of LMWOAs in soils are the litter, root exudates, and microbial metabolites, and a large amount of volatile fatty acids can also enter the forest soil during rainfall [7]. The LMWOAs’ concentration in forest soils is typically 10^−6^–10^−3^ mol/L. Although the LMWOAs’ content in the soil solution is low, LMWOAs can have a profound effect on the chemical and biological processes in forest soils and the growth of plants through complex mechanisms such as chelation and ligand exchange [8,9]. Research on forest LMWOAs has focused on forest soils and root exudates. Root exudates are one of the important sources of soil LMWOAs. The LMWOAs content in the root system is higher than that in the litter and soil. Organic acids secreted by roots promote the absorption of nutrients (N, P, Fe, and Ca) [10,11,12]. In addition, a few studies have found that many types of LMWOAs are produced during the decomposition and leaching of forest litter [13,14]. Due to different habitats, there are obvious regional differences in the types and contents of LMWOAs in the litter of different tree species. Few reports are available on the distribution characteristics of LMWOAs in evergreen broadleaf rhododendron forests.

Forest LMWOAs play an important role in the allelopathy of wood plants. Plant allelopathy refers to plants that affect neighboring plants or themselves by releasing chemical substances into the environment, resulting in beneficial or harmful mutual effects [15]. Additionally, studies have shown that long-chain fatty acids and organic acids are the main chemical species in the humus and soil surface of wild rhododendron forests and may be one of the most important factors for the natural regeneration of the forest, single plant species, and reverse succession [16]. The interaction between organic acids and soil microorganisms enhances the absorption of nutrients by plants [17]. The type and quantity of forest LMWOAs are affected by many factors, such as the forest soil nutrients, vegetation type, soil microorganisms, and other factors, but they are also affected by the seasons and rainfall [18].

**Figure 1:**
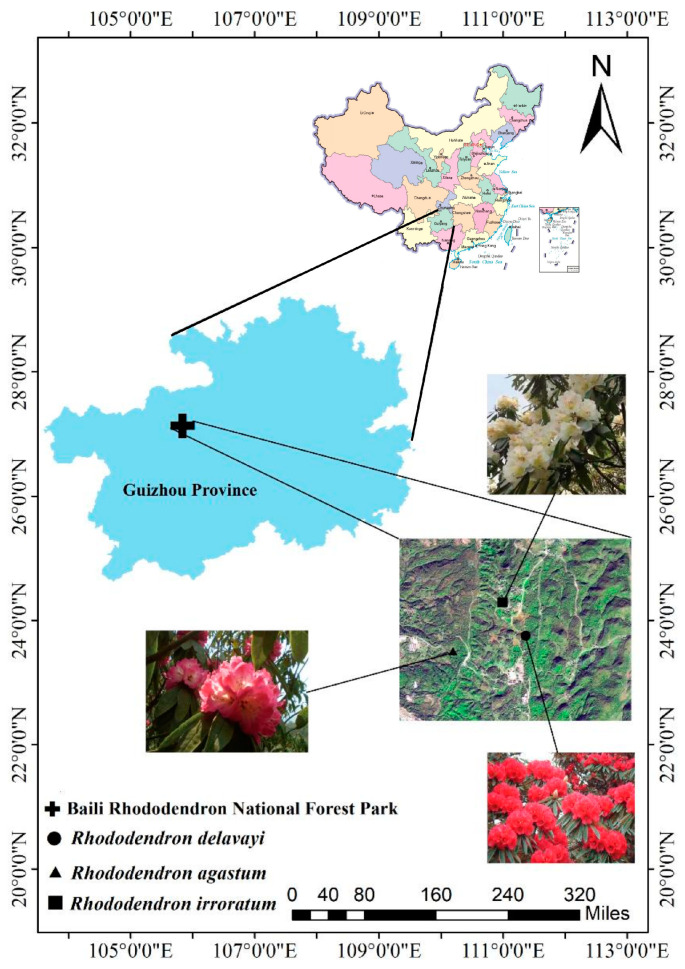
Distribution of the sampling points. Note: The map was drawn with reference to the Standard Map Service System (http://bzdt.ch.mnr.gov.cn/index.html (accessed on 3 December 2022), see the map number: GS (2019) 1675, Ministry of Natural Resources of the People’s Republic of China. The latitude and longitude show Guizhou Province and Baili Rhododendron National Forest Park).

This research takes the evergreen broadleaf wild rhododendron forest in Baili Rhododendron National Forest Park as the research object and studies the seasonal distribution characteristics and changes in the fresh leaves, litter, humus, and soil layer LMWOAs in the *Rhododendron delavayi* (RD), *Rhododendron agastum* (RA), and *Rhododendron irroratum* (RI) forests (Figure 1). The accumulation of the LMWOAs in the soils of the different forests and the differences in the distribution between species provide basic data for studying the ecological management and protection of wild rhododendron forests. The specific objective of this research is to address the seasonal variation of the organic acids present in wild rhododendron forests and to report the variation in the occurrence of the metabolites and the reasons for such variations.

## 2. Materials and Methods

### 2.1. Study Site

Baili Rhododendron National Forest Park (105°50′16″–106°04′57″ E, 27°10′07″–27°17′55″ N, elevation: 1060–2200 m) is located in the northwest of Guizhou Province, SW China [19]. It belongs to the plateau and hilly landform type. The climate is a mid-subtropical warm and humid monsoon climate. The annual mean temperature is 11.8 °C. The annual average relative humidity is 84%, which is wet in winter and dry in spring. The annual mean rainfall is 1000–1100 mm, with a wet season from May to October and a dry season from November to April [20]. Baili Rhododendron National Forest Park contains more than 40 species of wild rhododendrons, and it has the largest native wild rhododendron forests in China and the world [16].

### 2.2. Sample Collection and Processing

**Figure 2:**
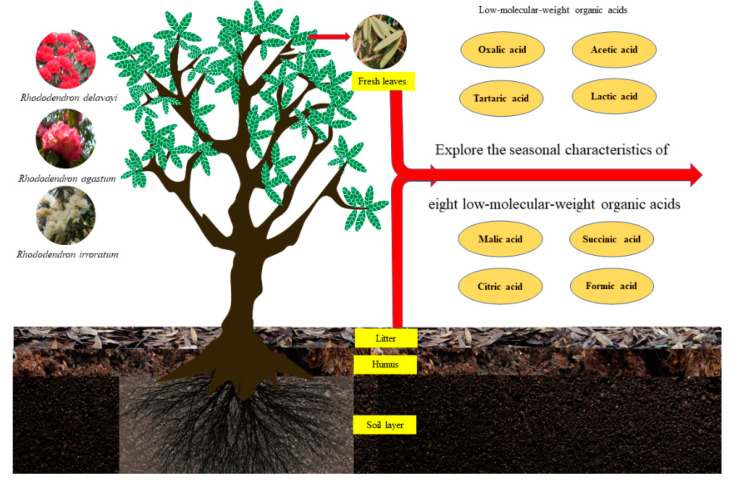
Experimental design showing the research object and research content.

The three dominant rhododendron forests in Baili Rhododendron National Forest Park (RD, RA and RI) were the research objects. Two plots in each species of forest (six plots) were used, and statistics were described for the basic conditions of the plots (Table 1). The plots were sampled in spring, summer, autumn, and winter. Representative trees were identified, and fresh leaves were collected from the first live branches in the east, south, west, north, and center directions [21]. Three 5 × 5 m plots were set up to collect litter, humus, and soil. The five-point method was used for stratified sampling. The soil samples were taken from the rhododendron forest soil at a depth of 0–10 cm (Figure 2). The samples were air-dried, ground, passed through a 100-mesh sieve, and stored (4 °C).

The determination method of the soil organic carbon (SOC), total nitrogen (TN), total potassium (TK), total phosphorus (TP), hydrolyzable nitrogen (HN), available potassium (AK), and available phosphorus (AP) was based on previous methods [22,23] and is shown in Table 2.

### 2.3. Instruments and Reagents

A high-performance liquid chromatography (HPLC) system (LC-10A, Shimadzu, Tokyo, Japan) and Capcell Pak C18 column (4.6 mm × 250 mm, 5 μm; Shiseido Co., Tokyo, Japan) were used. A high-speed refrigerated centrifuge was used (KDC-140HR, Anhui Zhongke Zhongjia Scientific Instrument Co., Ltd., Hefei, China), along with a constant-temperature culture oscillator (ZWY-211B, Shanghai Zhicheng Analytical Instrument Manufacturing Co., Ltd., Shanghai, China).

Oxalic acid, tartaric acid, formic acid, malic acid, lactic acid, acetic acid, citric acid, and succinic acid standards were obtained from Sigma (chromatographically pure; Sigma-Aldrich, St. Louis, MO, USA). Methanol (chromatographically pure, Tianjin Science and Europe Chemical Reagent Co., Ltd., Tianjin, China) and phosphoric acid (chromatographically pure, Chengdu Jinshan Chemical Reagent Co., Ltd., Chengdu, China) were obtained from commercial sources.

### 2.4. LMWOA Determination Methods 

The determination method was based on the method of Ali et al. [24]. Three repetitions of a 1 g sample were added to a 20 mL conical flask; for the soil, 1 g of the soil sample was mixed with 5 mL of 0.1% phosphoric acid solution; for the fresh leaves, litter, and humus, 1 g of the fresh leaves, litter, or humus sample was mixed with 10 mL of 0.1% phosphoric acid solution. The mixtures were vibrated in a constant-temperature culture oscillator at 25 °C for 24 h. The mixtures were allowed to stand for 10 min. The supernatant was placed in a 2 mL centrifuge tube and centrifuged at 6000 r/min for 10 min. The supernatant was passed through a 0.22 μm microporous membrane, and the filtrate was charged into a 2 mL centrifuge tube for HPLC.

### 2.5. Statistical Analysis

Data analysis was performed using SPSS 19.0 (SPSS Inc., Chicago, IL, USA), Microsoft Excel 2016 (Microsoft Inc., Redmond, WA, USA), and Origin 2019 software (Origin Software Inc., Northampton, MA, USA). A three-way analysis of variance was used to detect differences. A *p* value < 0.05 was considered significant. Correlation heatmap analysis (Spearman correlation) between the soil parameters and LMWOAs was performed using the OmicShare tools, a free online platform for data analysis (https://www.omicshare.com/tools) (accessed on 3 December 2022).

## 3. Results

### 3.1. Seasonal Dynamics of the LMWOAs in Three Forests

The contents of eight LMWOAs in the evergreen broadleaf rhododendron forests were affected by the tree species, samples, and season (Table 3). The effects of the species on the contents of eight types of LMWOAs showed nonsignificant differences. Except for tartaric and formic acid, the effect of the sample–season interaction was significant for all LMWOAs (*p* < 0.05). The effect of the species–season interaction on succinic acid was significant (*p* < 0.05).

### 3.2. Seasonal Characteristics of the LMWOAs in Three Forests

**Figure 3:**
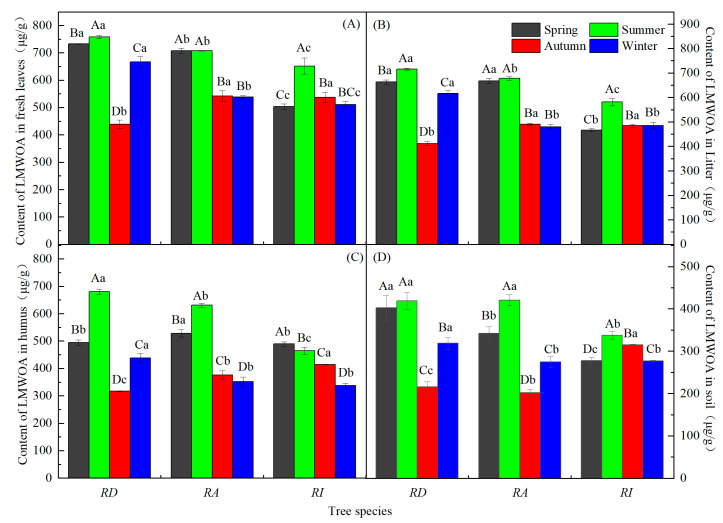
Seasonal dynamics of the LMWOAs in the different rhododendron forests. Note: (**A**–**D**) in the figure represent the seasonal dynamics of the LMWOAs in the fresh leaves, litters, humus, and soils in three rhododendron forests; different capital letters indicate the same azalea forest, and the LMWOAs’ content during the different seasons reached a significant level (*p* < 0.05); different lowercase letters indicate different azalea forests, and the LMWOAs’ content reached a significant level during the same season (*p* < 0.05). The values are the mean ± SE.

The LMWOAs’ content in various parts of the RD and RA soil varied with the seasons: summer > spring > winter > autumn, and most had significant changes (Figure 3; *p* < 0.05). Only the LMWOA content in the RD soil did not change significantly with the season (Figure 3D). The LMWOA content of the fresh leaves, litter, and humus changed seasonally in the RA (summer > spring > autumn > winter). The LMWOA content of the fresh leaves, litter, and soil in the RI forest changed seasonally, with an overall trend that increased during spring, peaked in summer, and was lowest during winter. The content of the humus LMWOAs in the RI forest decreased with seasonal changes (Figure 3C; *p* < 0.05). The LMWOAs content of the fresh leaves, litter, and humus in the different rhododendron forests were all significant during the summer (*p* < 0.05). The LMWOAs’ content in the different rhododendron forest soils were significantly different in spring and autumn (Figure 3D; *p* < 0.05). The order of the seasonal total LMWOAs’ content in different samples in the same rhododendron forests was fresh leaves > litter > humus > soil. In addition, except for the humus LMWOA content of the RI, the LMWOA content was the highest during the summer and lowest during the autumn/winter.

### 3.3. Differences and Characteristics of the LMWOAs in Three Forests

**Figure 4:**
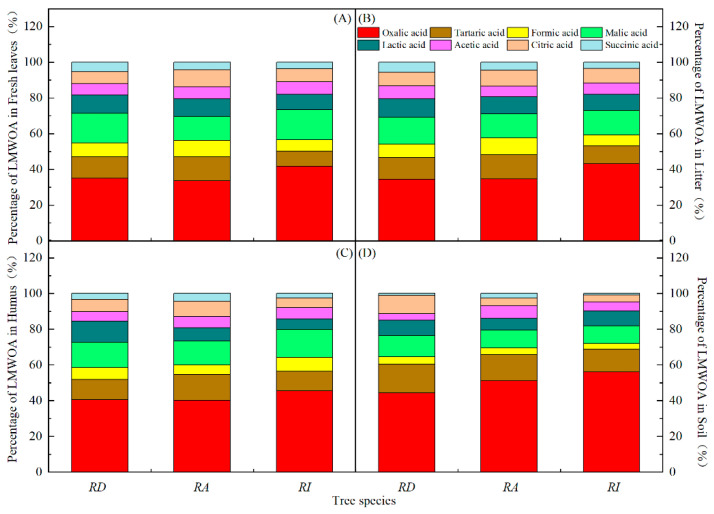
Composition and percentage of LMWOAs in the different rhododendron forests. Note: (**A**–**D**) in the figure represent the percentage of LMWOAs in the fresh leaves, litter, humus, and soil in different evergreen broadleaved rhododendron forests.

Eight types of LMWOAs were detected in the rhododendron forests (Figure 4). A greater distribution of LMWOAs was detected in the fresh leaves and litter of different species of rhododendron than in the rhododendron humus and soil. A greater distribution of various LMWOAs was observed in the RD litter than in the fresh leaves. The contents of oxalic, tartaric, and malic acid all exceeded 10%. Oxalic acid was the dominant LMWOA (33–57%), and the succinic acid content was the lowest (0.8–5.8%). The lactic and citric acid contents fluctuated widely in different samples of the rhododendron forests, but there was no obvious pattern. The proportions of the formic acid content in the fresh leaves, litter, humus, and soil of the rhododendron forests showed a downward trend. The proportion of acetic acid did not fluctuate much (3.7–7.1%). The main LMWOA was oxalic acid with an average content in the samples of 195.31 µg/g, followed by malic acid with an average content of 68.55 µg/g and tartaric acid with an average content of 59.82 µg/g. Succinic acid had the lowest content, and the average content in the samples was 18.40 µg/g.

**Figure 5:**
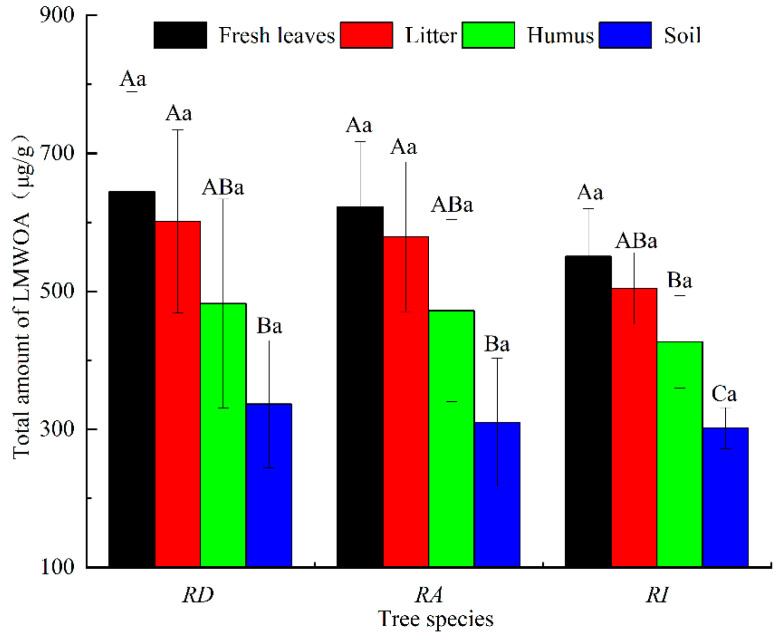
The total LMWOAs’ content in the different rhododendron forests. Note: Different capital letters represent significant content differences between different sampling positions of the same rhododendron (*p* < 0.05); different lowercase letters indicate that the LMWOAs’ content between the same sampling position in the different rhododendron forests was significant (*p* < 0.05). The values are the mean ± SE.

The order of the total amount of LMWOAs in the same part of the different rhododendron forests was RD > RA > RI (Figure 5). The LMWOAs in the RD were the highest, with an average content of 517.42 µg/g, followed by the RA with an average content of 495.18 µg/g and the RI with the lowest content of 445.18 µg/g. The order of the total amount of LMWOAs among the different samples in the same rhododendron forest was fresh leaves > litter > humus > soil. The LMWOA content of fresh leaves and litter in the RD was significantly higher than that in the soil (*p* < 0.05). The LMWOA content of the fresh leaves and litter in the RA was significantly higher than that in the soil (*p* < 0.05). Significant differences in the LMWOA content of the fresh leaves, humus, and soil were observed in the RI (*p* < 0.05).

**Figure 6:**
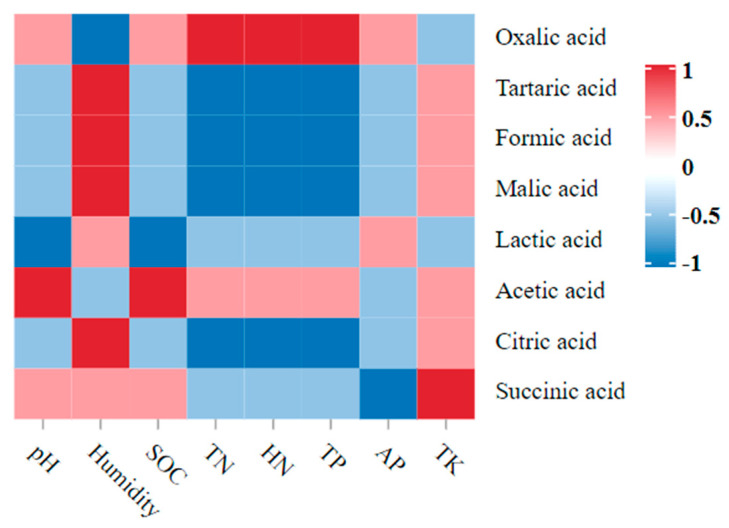
Dynamic correlation heatmap between the soil parameters and the LMWOAs. Note: Red indicates a positive correlation, and blue indicates a negative correlation.

The Spearman correlation between the soil parameters and the LMWOAs showed that the two indexes had obvious correlations (Figure 6). Among them, the pH value and the SOC were significantly positively correlated with acetic acid and significantly negatively correlated with lactic acid (*p* < 0.05). The humidity was significantly positively correlated with tartaric acid, formic acid, and malic acid, and it was significantly negatively correlated with oxalic acid (*p* < 0.05). The TN, HN, and TP were significantly positively correlated with oxalic acid, and they were significantly negatively correlated with tartaric acid, formic acid, malic acid, and citric acid (*p* < 0.05). The TK was significantly positively correlated with succinic acid (*p* < 0.05).

## 4. Discussion

### 4.1. Composition and Source of LMWOAs in the Forest

The composition and content of the LMWOAs secreted by different forest plants vary with plant species, inherent plant genetic characteristics, the natural environment, and cultivation measures [25,26]. In the present study, the LMWOAs’ content in three species of rhododendron forests were significantly different due to the influence of the species, samples, and seasons. LMWOAs are widespread in various parts of the forest. Oxalic, citric, lactic, formic, malonic, and succinic acid, as well as other LMWOAs have been identified in the roots, stems, leaves, litter, and humus of *Acer rubrum* L., *Cryptomeria japonica*, *Pinus koraiensis,* and other forest plants [16,27]. Our study showed that oxalic, tartaric, formic, malic, lactic, acetic, citric, and succinic acid were detected in the fresh leaves, litter, humus, and soils of the three species of rhododendron forests (RD, RA, and RI). Among them, oxalic acid had the highest content and accounted for the largest proportion, and oxalic acid was the dominant acid, which is consistent with previous studies [28]. Additionally, studies have shown that low P stress promotes increased levels of LMWOAs, particularly oxalic, citric, and malic acid, secreted by plants [29,30]. Therefore, the higher levels of oxalic, tartaric, citric, and malic acid in this study may be due to the low phosphorus content in the rhododendron soil environment (Table 2).

The sources of LMWOAs in the forest are complex. LMWOAs are continuously released into the soils from root exudates, microbial activities, and the decomposition of organic matter. Other secondary sources include atmospheric sedimentation [1]. The concentration of LMWOAs in soils is determined by the balance between production and degradation. Most LMWOAs are rapidly degraded by microorganisms in the soil [31]. The results of this experiment show that the order of the LMWOAs’ content in the different rhododendron forests from high to low was fresh leaves > litter > humus > soil, which is similar to previous research [32]. These results showed that the content of LMWOAs in the soil of the rhododendron forests was low.

### 4.2. Seasonal Characteristics of Forest LMWOAs

The season is a factor that affects the composition and content of forest LMWOAs. Previous research reported that the total amount of LMWOAs in *Cydonia oblonga* Miller leaves was significantly lower in October than in the two other months and decreased from June to August [33]. In this study, the LMWOAs’ content in the three rhododendron forests changed with the season, but most of the changes were not significant. The LMWOAs in the RD forest were significantly lower in autumn than during the other seasons. Research has reported that shikimate acid, a secondary metabolite of *Juniperus communis*, has the highest LMWOAs concentrations in summer, lower concentrations in spring and autumn, and undetectable levels in winter [34]. Our results confirm that the LMWOAs’ content in the three rhododendron forests was mostly highest in summer and lowest in autumn/winter. The LMWOA content in the humus of the RI forest was the highest in spring. The reason may be that the humus of the RI was very thick, forming a unique environment.

### 4.3. Potential Allelopathy of Forest LMWOAs

Allelopathic effects generally occur in the various organs of forest plants and various layers of the soil and can have varying effects on other plants. Allelopathy is caused by chemicals, and chemicals are typically secondary metabolites of plants, including organic acids, phenolic acids, flavonoids, terpenes, and other components [35]. Earlier research results showed that the plants and soil microorganisms in the rhododendron forest community and some chemicals extracted from the litter inhibited seed germination and seedling growth [36,37]. Previous studies concluded that phenolic acids and alcohols were the main chemical substances in the litter layer [16]. Previous research reported that plants and their ectomycorrhizas affect the content of chemicals (oxalic, citric, malonic, succinic, acetic, formic, and lactic acid) in forest soil [38]. The results showed that the three species of rhododendron forests had the largest accumulation of oxalic acid in the soil, which was the dominant LMWOA in the rhododendron forest. The LMWOA content was fresh leaves > litter > humus > soil. Therefore, the LMWOAs were more concentrated in the litter and humus layers. This may be why the regeneration of Baili Rhododendron National Forest Park is difficult, and the survival rate of the understory seedlings is low.

The season also affects the type and content of chemicals secreted by plants. Previous studies have shown that plants accumulate acids more, and the allelopathic activity during the summer is significantly stronger [39,40]. Additionally, the LMWOA content was higher in the summer than in the other seasons in our study.

## 5. Conclusions

The composition and content of the LMWOAs in the fresh leaves, litter, humus, and soil layers in three wild rhododendron forests were affected by the species, samples, and the season. Compared with the species, the seasons and samples had greater effects on the LMWOAs of rhododendron. Oxalic acid was the main LMWOA, followed by malic and tartaric acid, and succinic acid had the lowest content. The LMWOA content in the RD was the highest, and it was the lowest in the RI. The LMWOAs’ content of the three species of rhododendron forests was in the order of fresh leaves > litter > humus > soil layer. The LMWOAs in the forest mainly originate from plants and litter. The contents of oxalic, tartaric, malic, citric, acetic, lactic, succinic, and formic acid in the different rhododendron forests changed dynamically with the seasons. Therefore, according to the seasonal variation characteristics of LMWOAs in rhododendron forests, it will be the focus of future research to better understand the ecological functions of LMWOAs, such as reducing the pH value of the soil rhizosphere and producing allelopathic effects.

## Figures and Tables

**Table 1:** Basic situation of the forest plots.

General Situation	RD	RA	RI
Altitude (m)	1700	1658	1701
Latitude and longitude	105°51′49.76″ E 27°14′7.01″ N	105°51′7.94″ E 27°14′5.11″ N	105°51′39.54″ E 27°14′32.2″ N
Thickness of litter (cm)	3~5	2~4	4~7
Thickness of humus (cm)	10.5	16.5	20.4
Slope position	Mid-slope	Uphill	Uphill
Aspect	Southeast 115°	Southeast 128°	East 102°
Slope	22°	30°	30°

**Table 2:** Soil properties of the three rhododendron forests.

Soil Properties	RD	RA	RI
pH	4.31 ± 0.06	4.87 ± 0.08	4.80 ± 0.08
Humidity (%)	68.40 ± 2.66	62.50 ± 5.00	62.20 ± 1.96
SOC (g/kg)	81.27 ± 4.74	101.01 ± 6.09	97.94 ± 9.85
TN (g/kg)	2.15 ± 0.11	2.24 ± 0.16	4.00 ± 0.32
HN (mg/kg)	40.13 ± 1.39	43.54 ± 1.56	59.89 ± 9.68
TP (g/kg)	0.63 ± 0.04	0.67 ± 0.03	0.77 ± 0.05
AP (mg/kg)	0.42 ± 0.03	0.28 ± 0.02	1.02 ± 0.06
TK (g/kg)	3.38 ± 0.20	3.71 ± 0.12	2.84 ± 0.19
AK (mg/kg)	48.82 ± 2.29	44.93 ± 2.08	50.40 ± 4.73

The values are the mean ± SE, *n* = 3.

**Table 3:** Soil LMWOAs of three rhododendron forests.

Factors	df	Oxalic Acid	Tartaric Acid	Formic Acid	Malic Acid	Lactic Acid	Acetic Acid	Citric Acid	Succinic Acid
Species	2	0.639 ns	8.15 ns	1.504 ns	1.645 ns	2.052 ns	0.225 ns	1.184 ns	2.668 ns
Samples	3	49.379 ***	6.104 *	11.683 **	75.79 ***	16.133 **	18.968 **	6.914 *	15.083 **
Season	3	5.497 *	7.774 *	5.13 *	4.73 *	0.563 ns	2.871 ns	2.095 ns	10.033 **
Species × Samples	6	0.432 ns	0.704 ns	3.315 *	0.581 ns	1.337 ns	2.139 ns	3.112 *	3.83 *
Species × Season	6	1.391 ns	0.338 ns	1.55 ns	0.909 ns	0.777 ns	1.631 ns	2.075 ns	2.854 *
Samples × Season	9	3.226 *	1.246 ns	1.202 ns	3.087 *	4.82 **	5.036 **	4.002 **	3.759 *

The F ratio and *p* value of the three-way ANOVA were reported to understand the effects of the species, samples, and season on eight types of LMWOA content. ns: *p* > 0.05; * *p* < 0.05; ** *p* < 0.01; *** *p* < 0.001.

## Data Availability

The data presented in this study are available in insert article.
